# An intranasal vaccine comprising SARS-CoV-2 spike receptor-binding domain protein entrapped in mannose-conjugated chitosan nanoparticle provides protection in hamsters

**DOI:** 10.1038/s41598-023-39402-0

**Published:** 2023-07-26

**Authors:** Kairat Tabynov, Maxim Solomadin, Nurkeldi Turebekov, Meruert Babayeva, Gleb Fomin, Ganesh Yadagiri, Sankar Renu, Toktassyn Yerubayev, Nikolai Petrovsky, Gourapura J. Renukaradhya, Kaissar Tabynov

**Affiliations:** 1 International Center for Vaccinology, Kazakh National Agrarian Research University (KazNARU), Almaty, Kazakhstan; 2 Preclinical Research Laboratory with Vivarium, M. Aikimbayev National Research Center for Especially Dangerous Infections, Almaty, Kazakhstan; 3 T&TvaX LLC, Almaty, Kazakhstan; 4 School of Pharmacy, Karaganda Medical University, Karaganda, Kazakhstan; 5 Central Reference Laboratory, M. Aikimbayev National Scientific Center for Especially Dangerous Infections, Almaty, Kazakhstan; 6 Center for Food Animal Health, College of Food Agricultural and Environmental Sciences, The Ohio State University (OSU), Wooster, OH 44691 USA; 7 Vaxine Pty Ltd, 11 Walkley Avenue, Warradale, SA 5046 Australia; 8 Republican Allergy Center, Research Institute of Cardiology and Internal Medicine, Almaty, Kazakhstan

**Keywords:** Biotechnology, Immunology, Diseases

## Abstract

We developed a novel intranasal SARS-CoV-2 subunit vaccine called NARUVAX-C19/Nano based on the spike protein receptor-binding domain (RBD) entrapped in mannose-conjugated chitosan nanoparticles (NP). A toll-like receptor 9 agonist, CpG55.2, was also added as an adjuvant to see if this would potentiate the cellular immune response to the NP vaccine. The NP vaccine was assessed for immunogenicity, protective efficacy, and ability to prevent virus transmission from vaccinated animals to naive cage-mates. The results were compared with a RBD protein vaccine mixed with alum adjuvant and administered intramuscularly. BALB/c mice vaccinated twice intranasally with the NP vaccines exhibited secretory IgA and a pronounced Th1-cell response, not seen with the intramuscular alum-adjuvanted RBD vaccine. NP vaccines protected Syrian hamsters against a wild-type SARS-CoV-2 infection challenge as indicated by significant reductions in weight loss, lung viral load and lung pathology. However, despite significantly reduced viral load in the nasal turbinates and oropharyngeal swabs from NP-vaccinated hamsters, virus transmission was not prevented to naïve cage-mates. In conclusion, intranasal RBD-based NP formulations induced mucosal and Th1-cell mediated immune responses in mice and protected Syrian hamsters against SARS-CoV-2 infection but not against viral transmission.

## Introduction

To combat COVID-19 over 368 pandemic vaccine candidates have been developed, of which 170 have reached human clinical trials^1^. Eleven vaccines based on mRNA, inactivated, viral vector, and protein subunit platforms have been prequalified by the World Health Organization and used globally^2^. Mass immunization campaigns^3^ did not prevent the development of additional waves of disease due to new immune-escape virus mutations^4,5^. Booster immunizations has been implemented to try and maintain vaccine effectiveness^6^. Different types of vaccines have also been tested in mixed combinations in a heterologous manner^7,8^. Unfortunately, such approaches have had very limited impact on SARS-CoV-2 virus spread and have been associated with rare but severe adverse reactions^9^. Therefore, a search for new COVID-19 vaccine approaches, particularly ones that induce improved mucosal immunity, are warranted.

We developed a subunit SARS-CoV-2 vaccine for intranasal administration, containing spike protein receptor-binding domain (RBD) entrapped in mannose-conjugated chitosan nanoparticle (NARUVAX-C19/Nano). This platform is intended to induce both systemic and local mucosal immune responses. Anti-RBD antibodies block the interaction of the virus with the angiotensin-converting enzyme 2 (ACE2) cell receptor and neutralize the virus by preventing its invasion^10,11^. Intranasal/intrapulmonary delivery of antigen induces mucosal immunity including production of sIgA antibodies, which are the first line of defense against respiratory pathogens^12^. In addition, intranasal vaccination is a needle-free noninvasive method which eliminates several issues (local pain and discomfort at injection site, increased vaccine cost, need of trained person for vaccination, and fear of injection)^13^. Intranasal immunization provides a large absorption area and does not expose antigens to extreme pH conditions^14^. Nanoparticle (NP)-based vaccine approaches protect the antigens from premature degradation, increase stability, and ensure targeted delivery of the immunogen to antigen-presenting cells (APC)^15,16^. Chitosan is a natural carbohydrate polymer, biocompatible, bioavailable, and forms highly positively charged NPs which electrostatically interact with negatively charged sialic acid on mucus and epithelial surfaces making it a strong mucoadhesive vaccine vehicle^17^. Surface labeling NP with mannose, a calcium-dependent (type C) mannose receptor of the lectin family assists binding to dendritic cells and macrophages^18^, providing a pronounced adjuvant effect^17,19,20^. To enhance the T-cell response of the NP-formulation, CpG55.2 oligonucleotide adjuvant, a human toll-like receptor 9 agonist^21^ (Vaxine Pty Ltd, Adelaide, Australia) was included in the final vaccine composition. The NP-vaccine formulations were evaluated for immunogenicity in mice and protective efficacy in hamsters, by comparison to a traditional intramuscular RBD protein vaccine formulated with an aluminum hydroxide (alum) adjuvant.

## Materials and methods

### Virus, biosafety and bioethics

The virus strain hCoV-19/Kazakhstan/KazNAU-NSCEDI481/2020 of wild-type SARS-CoV-2 with D614G mutation in spike protein was used. This virus was isolated at the Aikimbayev National Research Center for Especially Dangerous Infections (NSCEDI) in June 2020 from the nasopharyngeal swab of a 45-year-old COVID-19 patient in Almaty, Kazakhstan (GISAID, #EPI_ISL_514093). The virus was grown in vitro as described previously^22^. In this study, we used the 3rd passage virus which had an infectious titer of 6.2 log_10_ TCID_50_/mL.

All the SARS-CoV-2 experiments were performed in NSCEDI’s BSL-3 and ABSL-3 laboratories. This study was conducted in accordance with national and international laws and guidelines for the handling of laboratory animals. The protocol was approved by the Institutional Committee on the Keeping and Use of Laboratory Animals of the NSCEDI (Protocol No. 4, dated September 22, 2020). All methods are reported in accordance with the ARRIVE guidelines (https://arriveguidelines.org) for the reporting of animal experiments.

### Vaccine preparation

Spike protein RBD [Gln321-Ser591] expressed in HEK293 cells with purity > 95% by SDS-PAGE and endotoxin < 1.0 EU per μg protein by the LAL method was purchased from a commercial source (ABP Biosciences, USA). The SARS-CoV-2 Spike-RBD vaccine formulation was prepared using mannose-conjugated chitosan nanoparticles (NP-vaccine) by a standard ionic gelation method, as described previously^23^. The NP morphology, antigen loading efficiency, and size distribution was determined using appropriate methods. The vaccine formulation was lyophilized and stored at − 20 °C until use. Resuspension of the NP-vaccine was carried out with PBS to the desired volume. In one formulation, CpG55.2 oligonucleotide (Vaxine Pty Ltd, Australia) was added at the same time as entrapping RBD to obtain a TLR9-adjuvanted NP-vaccine formulation (NP-CpG vaccine). The intramuscular vaccine (RBD-Alum) used for comparison was prepared by mixing RBD protein with alum (Alhydrogel® adjuvant 2%, InvivoGen, CA, USA). All vaccine formulations were kept sterile and contained < 2 EU/dose endotoxin. The details of vaccine content is provided in Table 1.

**Table 1:** Vaccine formulations and routes of administration.

Formulation	RBD protein concentration per 50 µL (dose) of vaccine, µg	Adjuvant concentration per 50 µL (dose) of vaccine	Method of administration
NP vaccine	5, 2.5, 1.25	–	IN
NP-CpG vaccine	5, 2.5, 1.25	10 µg (CpG)	IN
Antigen alone	5	–	IN
Alum vaccine	5	1 mg (Alum)	IM

NP, nanoparticles; IN, intranasally; IM, intramuscularly.

### Particle size determination

The particle size distribution and mean diameter of NP-formulations were assessed in aqueous dispersions with proper dilution using the dynamic light scattering (DLS) technique or photon correlation spectroscopy (DLS Zetasizer Nano ZSP; Model-ZEN5600; Malvern Instruments Ltd., Worcestershire, UK) in disposable polystyrene cuvettes (Model DTS0012; Malvern) at 25 °C. All the readings were taken in triplicate at different time intervals and for independent experiments with He–Ne laser 633 nm and with avalanche photodiode detector (APD). The average of 3 readings (each reading = 30 runs) was reported as the actual particle size.

### Scanning electron microscopy

The morphology of the nanoparticles was determined by using scanning electron microscopy. NP suspension (5 μL) was placed on the cleaned silicon wafer chip (SPI Supplies, USA) (Cat no. 4136SC-AB) and then on aluminum stubs, air dried in fume hood for 60 min and kept overnight under vacuum. Samples were coated with platinum for up to 30 nm thickness in the Q150T plus sputter coater (Quorum Technologies, UK) and imaging was done on the Hitachi SU5000 Field emission scanning electron microscope.

### Entrapment efficiency

The protein entrapment efficiency in mannose-conjugated chitosan NP was estimated by an indirect method by determining difference between protein amount found in the vaccine formulation supernatant and initial amount used. The amount of protein present in the supernatant was measured using the micro-BCA protein assay kit (Biorad, USA). Entrapment efficiency (%) = [(RBD protein added − Free “unentrapped RBD protein”)/RBD protein added] * 100.

### Vaccination and immune response analysis in mice

Four to-six-week-old SPF (specific pathogen-free) female BALB/c mice obtained from the NSCEDI Laboratory Animal Breeding Facility were used in the vaccine trial. Animals were placed in ventilated cages with HEPA filters (Allentown, USA) for 7 days prior to the experiment for acclimatization. Mice were immunized by intranasal route with NP-vaccine formulations, antigen alone, and PBS intranasally under ketamine (100 mg/kg) and xylazine (10 mg/kg) anesthesia in a volume of 50 µL twice at 21-day interval. This volume of intranasal vaccine in a mouse typically results in both nasal and intrapulmonary delivery of the antigen^24^. The Spike RBD in Alum vaccine was administered by intramuscular route. At 21 days post prime (n = 7/group) and booster (n = 7/group) vaccination, blood samples were collected from the orbital venous sinus to determine RBD-specific sIgA, IgG antibodies and IgG antibody isotypes (IgG1 and IgG2a), RBD-ACE2 blocking antibodies and virus neutralizing titers against a wild-type SARS-CoV-2 (D614G).

At day 21 after booster (n = 3/group) vaccination, mice were euthanized by cervical dislocation under ketamine-xylazine anesthesia and the spleen collected under aseptic conditions to evaluate the cellular immune response and the lungs collected for determining RBD-specific sIgA antibodies.

### Determination of humoral and cellular immune responses

The levels of anti-RBD sIgA, IgG, IgG1, and IgG2a antibodies were determined by enzyme linked immunoassay (ELISA) as previously described^22^. SARS-CoV-2 Surrogate Virus Neutralization Test (sVNT) Kit (L00847; GenScript, Piscataway, USA) was used to detect antibodies that inhibit RBD binding to the ACE2 cell receptor, according to the manufacturer’s instructions. The inhibition percentage of the sample was calculated as (1-Average OD of the sample/Average OD of the negative control) × 100%. A sample with an inhibition percentage < 30% was considered “negative” and ≥ 30% was considered “positive” for SARS-CoV-2 neutralizing antibodies. The following neutralizing antibody values were determined according to the level of inhibition: low (30–59%), medium (60–89), high (90 ≤). Determination of viral neutralizing antibodies was performed as previously described^22^ using 1000 TCID_50_ of wild-type SARS-CoV-2 (D614G). The neutralizing antibody titer was the highest dilution of serum that inhibited the cytopathic effect in 100% of wells.

Cellular immunity was determined by measuring cytokine production in a suspension of splenocytes in response to restimulation for 72 h with 5 µg RBD protein including IL-2 (#B320273), IFN-γ (#B307222), IL-4 (#B320413), IL-10 (#B311304), IL-5 (#B317463), IL-6 (#B321215), IL-17A (#B303513), TNF-α (#B306271), using ELISA MAX™ Deluxe Set Mouse kits (BioLegend), as previously described^22^. Cytokine production data were presented as the difference (delta) of cytokine concentrations (pg/mL) between samples with and without RBD protein stimulation.

### Hamster vaccination and protective efficacy evaluation

Six- to eight-week-old male Syrian hamsters obtained from the NSCEDI Laboratory Animal Breeding Facility were used in the vaccine trial. Animals were placed in ventilated cages with HEPA filters for 7 days prior to the experiment for acclimatization. Hamsters were immunized with NP-vaccine formulations (NP vaccine and NP-CpG vaccine, n = 6/group) containing RBD protein 5 µg/dose or PBS (control group, n = 6) intranasally in a 100 µL volume under ketamine (80 mg/kg) and xylazine (8 mg/kg) anesthesia twice at 21-day intervals. For comparison, an RBD-alum vaccine containing 5 µg/100 µL of RBD protein was administered intramuscularly.

At day 21 after booster vaccination, hamsters were intranasally challenged with SARS-CoV-2 at a dose of 1 × 10^4^ TCID_50_/100 µL under ketamine-xylazine anesthesia and observed for 7 days post infection with daily measurement of body weight. On days 3 and 7 post challenge, half of the animals (3/6) from each group were euthanized and nasal turbinates and lung samples collected. Three lobes of the right lung from each animal were fixed in 10% formaldehyde for histopathological studies. Two lobes of the left lung were homogenized in 1 mL DMEM using a TissueLyser II instrument (QIAGEN) at 300 vibrations/min for 60 s, the supernatant after centrifugation (5000 g for 15 min at 4 °C) was collected and stored at − 70 °C for later determining the viral titer.

### Virus transmission assessment

For all groups of hamsters on the 2nd day of the challenge, two naïve animals were placed in the same cage for a 1-day contact period, and then separated into clean cages, where they were kept in isolation for an additional 4 days with daily monitoring of weights. They were then euthanized to assess viral load in the nasal turbinates and lungs, and pathological changes in the lung.

### Analysis of viral titers and histological evaluation

Virus titers in the tissue homogenates were determined as previously described^22^, being determined using the Reed and Mench method expressed in log_10_ TCID_50_/0.2 mL. Histological analysis of hamster lungs was performed as previously described^22^. Each slide was quantified based on the severity of histologic changes, including interstitial pneumonia, alveolitis, bronchiolitis, alveolar destruction, interstitial infiltration, pulmonary hemorrhage, and peribronchiolar inflammation. Based on the previously described method^22,25^, the assessment score was as follows: 4 points—extremely severe pathological lung changes; 3 points—severe pathological lung changes; 2 points—moderate pathological lung changes; 1 point—mild pathological lung changes; 0 points—no pathological changes.

### Statistical analysis

The GraphPad Prism 9.0.0 computer software (GraphPad Software, San Diego, CA, USA) was used for plotting and statistical analysis of experimental data. Differences in antibody levels, cytokine production, viral load in respiratory organs and oropharyngeal swabs, weight dynamics, and pathological changes in the lungs between animal groups were assessed using Dunnett’s multiple comparisons test or Tukey’s multiple comparisons test. The limit of viral titer detection was 0.7 log_10_ TCID_50_/0.2 mL. The limit of detection for neutralizing antibodies was 3.0 log_2_. Geometric mean titers with 95% confidence interval were calculated for neutralizing antibody analysis. For all comparisons, *P* < 0.05 was considered significant. All error bars in the graphs represent the standard error of the mean.

## Results

### Nanoparticle characteristics

**Figure 1:**
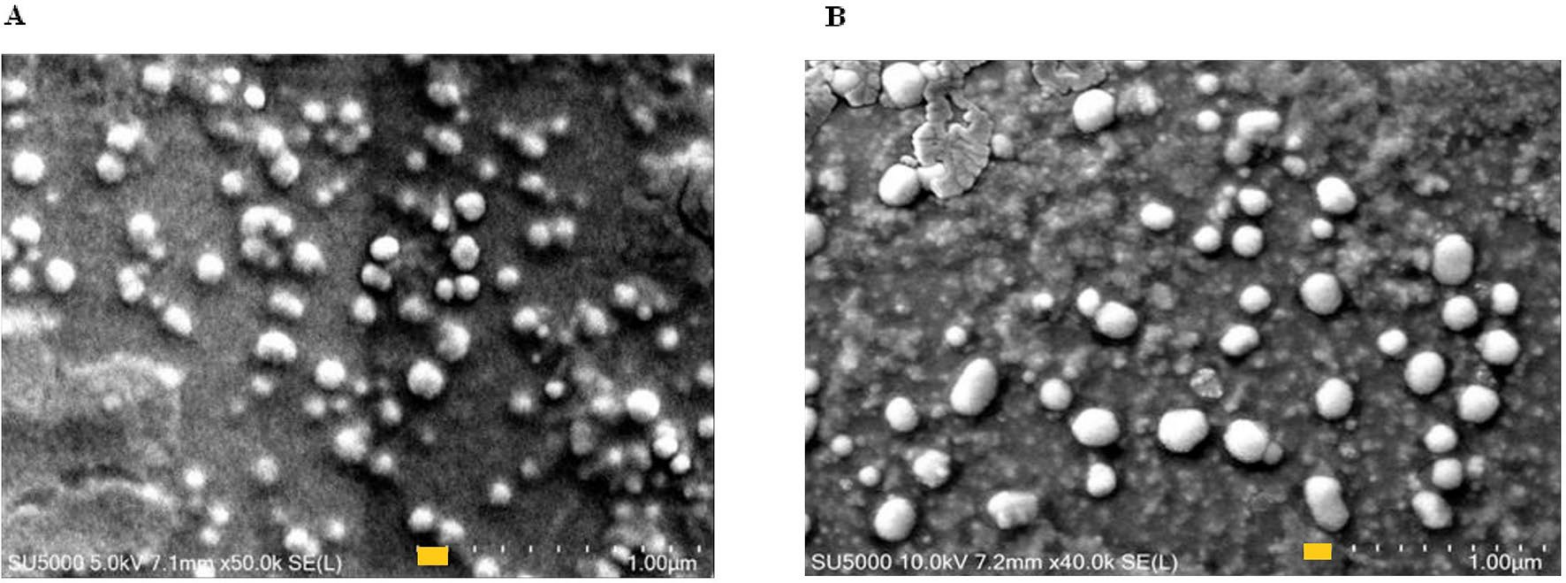
Scanning electron microscope field emission analysis of (**A**) mannose-conjugated chitosan nanoparticles and (**B**) mannose-conjugated chitosan nanoparticles entrapping RBD protein. For size comparison display a yellow bar notation measuring 100 nm.

The average size of mannose-conjugated chitosan particles and RBD protein-entrapped mannose-conjugated chitosan particles was 180 ± 12 nm and 290 ± 18 nm, respectively. Scanning electron microscopy showed that the particles were discrete, spherical, and regular in shape (Fig. 1). The uptake rate of RBD protein into mannose-conjugated chitosan nanoparticles was measured to be 66.8%.

### Intranasal NP-vaccine induced RBD-specific sIgA antibodies in mice

**Figure 2:**
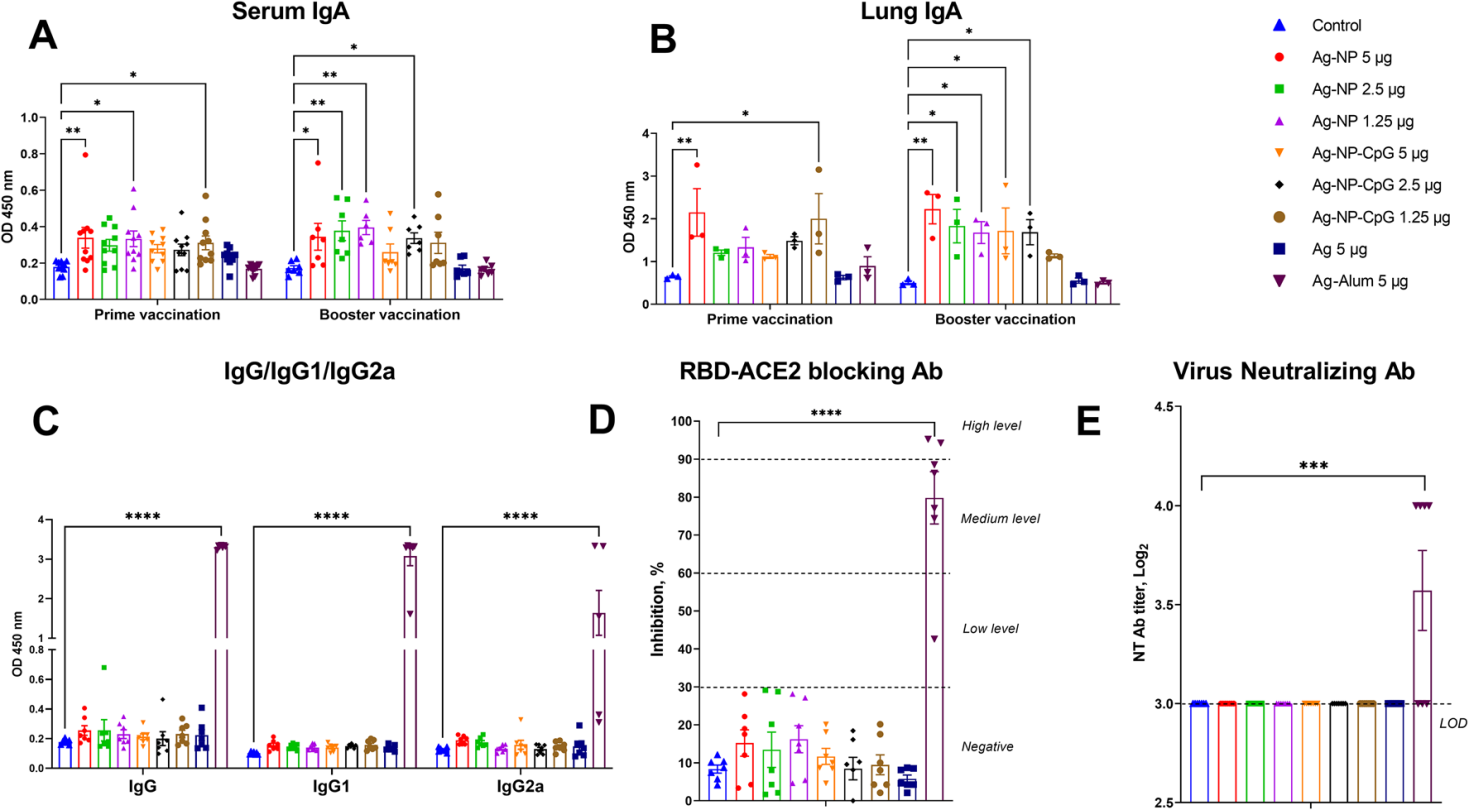
Antibody response in BALB/c mice after vaccination. RBD-specific serum (**A**) and lung (**B**) IgA in mice at 21 days after prime and booster intranasal immunization with RBD-based nanoparticle vaccine formulations and intramuscular immunization with alum-adjuvanted RBD vaccine. Levels of IgG, IgG1, IgG2a (**C**) and RBD-ACE2 blocking antibodies (**D**) as well as viral neutralizing titers (**E**) in mice after booster immunization. Mannose-conjugated chitosan-nanoparticle (NP)-based vaccine formulations, including those with CpG adjuvant (NP-CpG), were administered twice in doses containing 5, 2.5, and 1.25 µg RBD protein. For comparison, studies included antigen alone group (Ag) with an intranasal immunization of 5 µg RBD protein/dose, an intramuscular alum adjuvanted (5 µg RBD/dose) group, and a control group (PBS). Antibody levels are presented as optical density at 450 nm. A sample with an inhibition percentage < 30% was considered “negative” and ≥ 30% was considered “positive” for SARS-CoV-2 neutralizing antibodies. The following neutralizing antibody values were determined according to the level of inhibition: low (30–59%), medium (60–89), high (≥ 90). Viral neutralizing levels with the wild-type of SARS-CoV-2 (D614G) virus are presented as geometric mean titers with 95% confidence intervals (**E**). Differences in antibody levels between animal groups were assessed using Dunnett’s multiple comparisons test. *P* < 0.05 was considered statistically significant. *P < 0.05, **P < 0.01, ***P < 0.001, and ****P < 0.0001.

Intranasal/intrapulmonary (IN/IPM) immunization of mice with one or two doses of NP-vaccine formulations induced measurable RBD-specific sIgA antibodies in serum (Fig. 2A) that were even more prominent in the lung (Fig. 2B). NP-vaccine formulations induced significantly higher sIgA antibody levels compared to controls, including the RBD-alone group. However, booster immunization did not significantly increase either serum or lung sIgA antibodies in any NP-vaccine groups. Intranasal NP-vaccine formulations did not induce detectable levels of serum anti-RBD IgG, IgG1, IgG2a, RBD-ACE2 blocking or virus-neutralizing antibodies (Fig. 2C–E). In contrast, after the second immunization the IM-administered alum-adjuvanted spike-RBD vaccine induced serum anti-RBD IgG, IgG1, and IgG2a (Fig. 2C) as well as RBD-ACE2 blocking (Fig. 2D) and SARS-CoV-2 neutralizing antibodies (GMT 15.7, Fig. 2E). Notably, a mucosal anti-RBD sIgA response was not detectable in the IM-immunized mice.

### Intranasal NP-vaccine elicits a Th1-polarized cellular immune response

**Figure 3:**
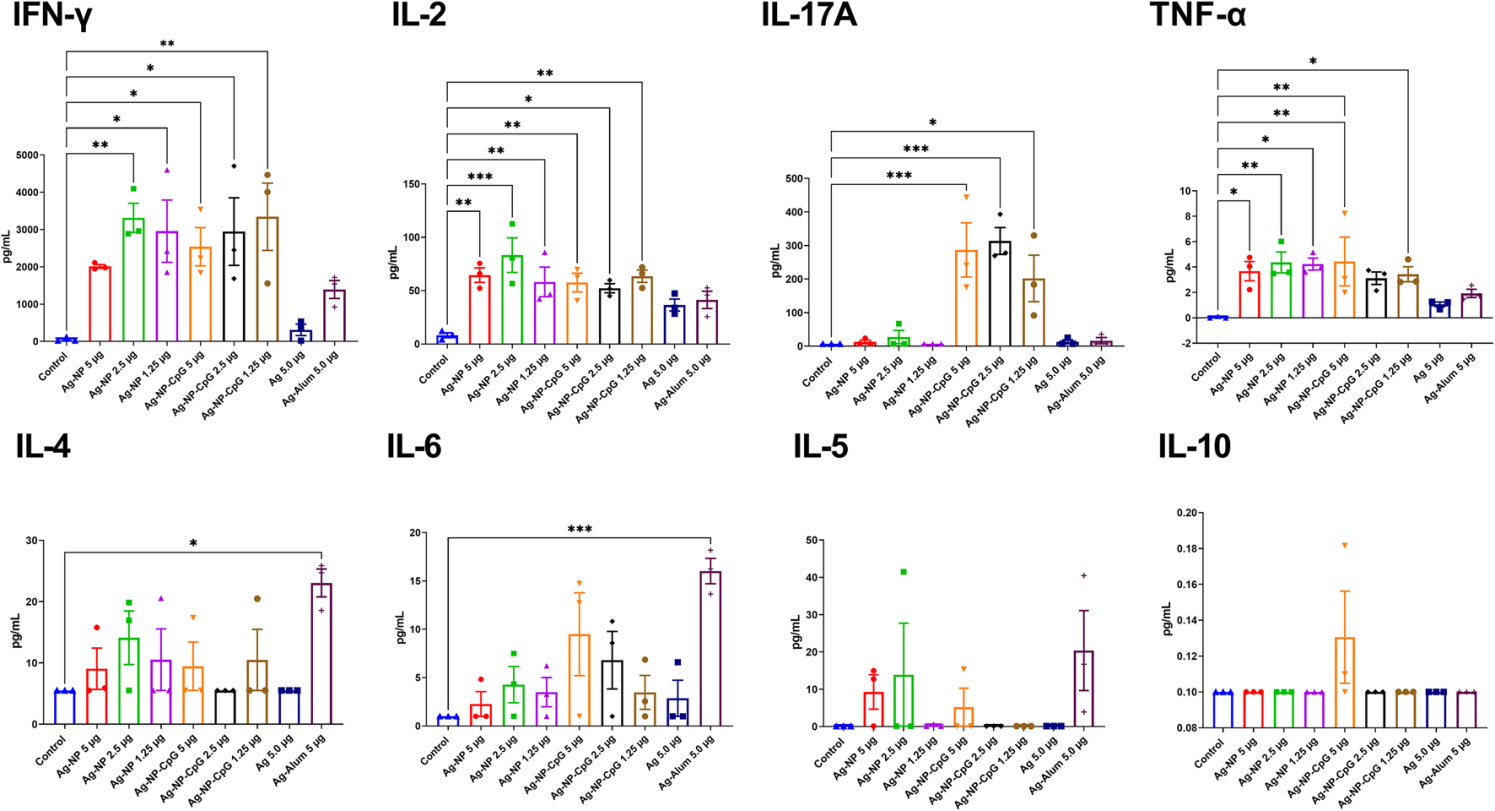
Antigen-specific cytokine production in the splenocyte suspension of BALB/c mice at day 21 post booster intranasally immunization with RBD-based NP-vaccine formulations and intramuscular immunization with Alum adjuvanted vaccine. Mannose-conjugated chitosan-nanoparticle (NP)-based vaccine formulations with and without including CpG adjuvant (NP-CpG), were administered in doses containing 5, 2.5, and 1.25 µg RBD protein. For comparison included antigen alone group (Ag) delivered intranasally with 5 µg RBD protein/dose, an Alum adjuvanted (5 µg RBD/dose) vaccine group administered intramuscularly, and a control group (PBS). Cytokine production data were presented as the difference (Delta) of cytokine concentrations (pg/mL) between samples with and without RBD stimulation. Differences between animal groups were assessed using Dunnett’s multiple comparisons test. *P* < 0.05 was considered statistically significant. *P < 0.05, **P < 0.01, ***P < 0.001.

Evaluation of production of eight cytokines in response to restimulation of mouse splenocytes with RBD protein showed that the intranasal NP-vaccine induced memory T cells of a Th1 phenotype, secreting IFN-γ, IL-2, and TNF-α, in response to antigen stimulation (Fig. 3). Only the intranasal NP-CpG vaccine induced significant IL-17 recall responses consistent with a Th17 response. In contrast, the intramuscular RBD-alum vaccine induced memory T cells that produced significantly higher levels of Th2 cytokines including IL-4 and IL-6.

### Intranasal NP-vaccines provides protection against wild-type SARS-CoV-2 (D614G) infection in hamsters, but did not block viral transmission

**Figure 4:**
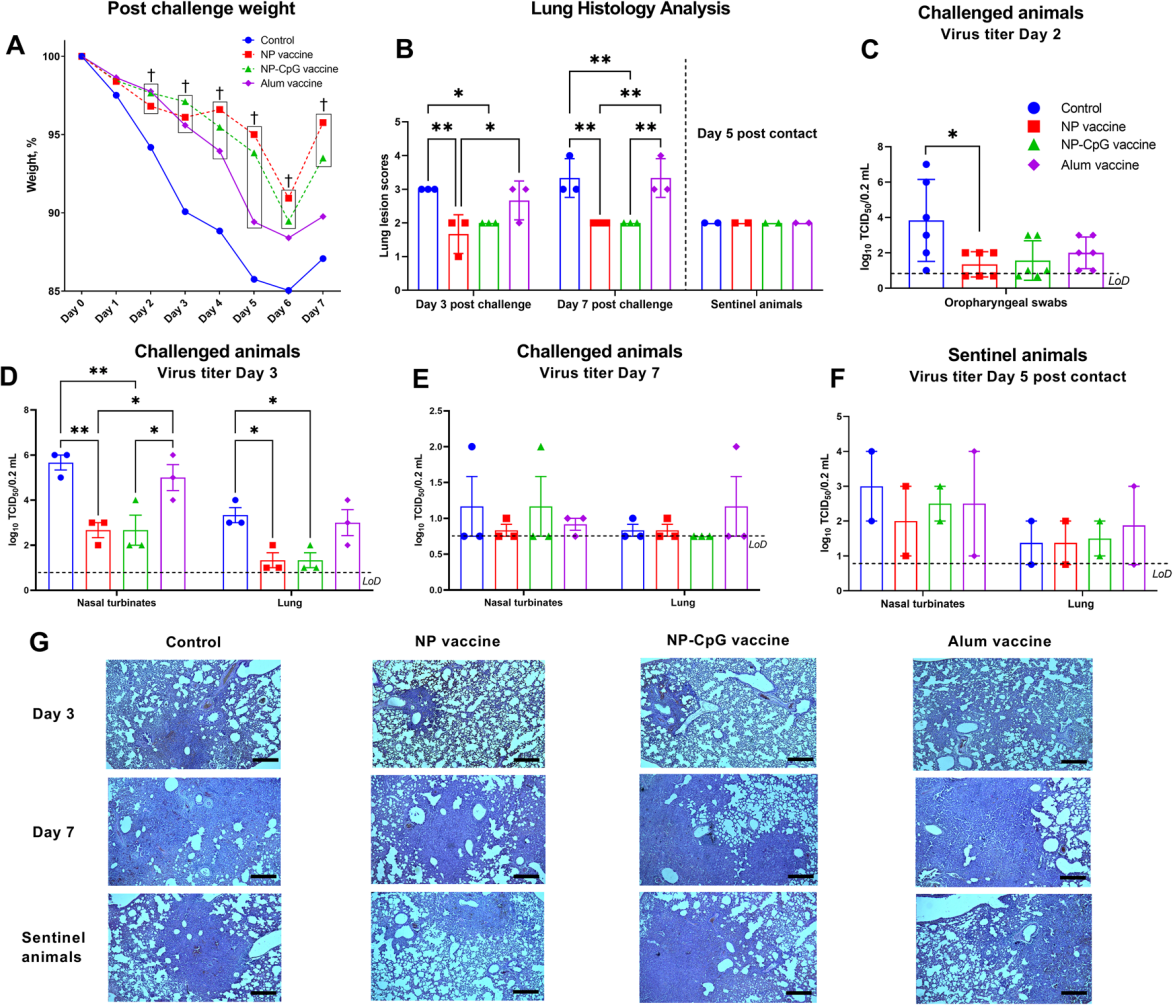
Efficacy of RBD-based NP-vaccine in Syrian hamsters for protection against wild-type SARS-CoV-2 (D614G) infection and virus transmission. Mannose-conjugated chitosan-nanoparticle (NP)-based vaccine formulations, including the CpG adjuvant (NP-CpG vaccine), were administered with a dose containing 5 µg RBD protein. For comparison included intramuscular alum-adjuvanted (5 µg RBD/dose) group, and control group (PBS). Animals were intranasally challenged with SARS-CoV-2 and the following parameters were studied: Changes in body weight (**A**); Viral load in oropharyngeal swabs (expressed as log_10_ TCID_50_/0.2 mL) on day 2 (**C**) after challenge; Viral load in nasal turbinates and lungs on day 3 (**D**) and day 7 (**E**) after challenge; Viral load in nasal turbinates and lungs of sentinel animals on day 5 (**F**) post co-housing with infected animals; Pathological changes in the lungs of animals on days 3 and 7 after challenge, as well as sentinels on day 5 after co-housing with challenged animals by histological analysis (**B**,**G**). Photographs were taken at × 40 magnification. Scale bars are 500 µm. Differences between the animal groups were assessed using Dunnett’s multiple comparisons test (**A**,**C**) or Tukey’s multiple comparisons test (**B**,**D**). *P* < 0.05 was considered statistically significant. *P < 0.05, **P < 0.01. ^†^*P* = 0.03 to ˂0.0001 compared to the control group.

Protective efficacy against clinical disease was evaluated clinically by recording changes in body weight for 7 days post challenge infection (Fig. 4A). Both vaccinated and unvaccinated hamsters had a steady decrease in body weight up to 6 days post-infection. However, NP-vaccine immunized animals had a significantly reduced weight loss over the study period compared to control groups. Weight loss at peak was 9–10.5% in vaccinated animals and 15% in control animals. In the NP-vaccine groups in the body weight loss was slightly less than the alum-adjuvanted intramuscular vaccine group and was significantly better than the control group at 2–5 days post challenge.

Virus load was determined by measuring the infectious viral titer in oropharyngeal swabs of hamsters at day 2 post challenge (Fig. 4C), as well as in nasal turbinates and lungs of euthanized animals at day 3 (Fig. 4D) and day 7 (Fig. 4E) after infection. The virus load in the oropharyngeal swabs of vaccinated hamsters was lower than in the control group, and this difference was significant for the NP-vaccine group. Virus was detected in the respiratory organs of all hamsters, with the highest titers on day 3 post challenge. However, the viral load in NP-vaccine immunized animals in nasal turbinates and lungs was significantly lower in comparison not only to the control group, but also to the alum-adjuvanted vaccine group. During the period of observation, the virus titers on day 3 in the nasal turbinates was much higher than in the lungs, suggesting that sterilizing immunity for SARS-CoV-2 in the upper respiratory tract could be hard to achieve.

In the lungs of all infected hamsters, including the vaccinated ones, the presence of classical signs of acute respiratory distress syndrome (ARDS) manifested by SARS-CoV-2 infection was confirmed. On day 3, the lungs of hamsters had signs of the exudative phase of ARDS, which by day 7 changed to the fibroproliferative phase of ARDS (Fig. 4G). Comparative morphological characterization of lungs in terms of lesions was significantly lower in both intranasal NP-vaccine groups compared IM RBD-alum and the unimmunized control group (Fig. 4B). Interestingly, in the IM alum-RBD group, despite their high serum antibody levels, the amount of lung damage was as high as in the control group.

None of the vaccines blocked virus transmission from challenged animals to naïve sentinels. Sentinels in all groups showed weight loss (3.9–4.8% at 5 days post co-housing, data not shown) and the presence of virus (Fig. 4F) in the nasal turbinates (in 2/2 of each group) and lungs. All the sentinel hamster groups had similar level of lung damage as per histological analysis (Fig. 4B).

## Discussion

In this study, we sought to deliver RBD antigen using a nanoparticle delivery system with CpG55.2 oligonucleotide adjuvant (TLR-9 agonist) to achieve a novel needle-free, mucosally delivered, COVID-19 vaccine. Currently, only eight of the 170 vaccines that are under clinical studies are administered mucosally and, of those, only two are subunit vaccines^1^ with the other intranasal vaccines being virus vector based. A NP-based vaccine formulation derived from mannose-conjugated chitosan polymer was prepared at Ohio State University (OSU, USA) using an optimized strategy previously used for swine influenza virus^19,20^ and avian salmonellosis^26^ NP vaccines. Our candidate NP vaccine called NARUVAX-C19/Nano is listed by the WHO as a Covid-19 vaccine under preclinical studies^1^. Based on previous data^27–29^ on the effectiveness of CpG oligonucleotide adjuvant in enhancing the immunogenicity of several intranasal vaccines, a NP-vaccine formulation containing the adjuvant CpG55.2 (Vaxine Pty Ltd, Adelaide, Australia) was tested in our NP formulation. This adjuvant was included in an intramuscular subunit SARS-CoV-2 vaccine called Covax-19/SpikoGen® which demonstrated safety and efficacy in Phase III clinical trials and has received approval for emergency use in Iran^30–32^.

In this study, intranasal administered NP-vaccine formulations elicited a different immunogenicity profile from that of alum-adjuvanted RBD given IM. Neither of the intranasally administered NP-vaccine formulations induced measurable serum neutralizing antibodies, but instead induced mucosal sIgA and splenocytes T cell responses. This feature of our NP vaccine is a cardinal difference from other intranasal subunit RBD- or Spike-based vaccines conjugated with diphtheria toxoid (EcoCRM®)^33^ or outer membrane vesicles (OMVs) from *Neisseria meningitidis*^34^, respectively, which induced both systemic neutralizing antibodies and local IgA responses. However, despite the absence of serum neutralizing antibodies^10,11^, our NP-vaccine formulations provided significant protection of vaccinated hamsters against SARS-CoV-2 infection. The NP-vaccine significantly reduced the challenge virus load in both the upper and lower respiratory tract, and, most importantly, reduced lung damage in hamsters after challenge to a greater extent than the intramuscular vaccine group. Protection with NP-vaccine formulations was likely due to both anti-RBD sIgA and T cell immunity, consistent with findings of others^35–37^. However, given that sIgA had no neutralizing effect in the RBD-ACE2-blocking test as well as the viral neutralization reaction, it is likely that the role of cellular immunity with NP-vaccine formulation was predominant in providing protective efficacy^36^. In the available literature, we did not find data on NP based vaccines that form exclusively IgA antibodies. However, it should be noted that a similar phenomenon was observed previously during the development of a cold-adapted modified-live equine influenza virus vaccine. This vaccine, when administered intranasally to horses, also did not induce the formation of serum antibodies (indicated by the HI reaction and ELISA). However, it did induce the production of secretory IgA antibodies in nasal swabs and a cellular immune response^38^. Subsequently, this vaccine, when administered intranasally once or twice, provided significant clinical and virological protection against homologous and heterologous equine influenza virus for 12 months post-vaccination^39^.

Although the addition of CpG to the NP-vaccine formulation induced increased production of IL-17, a Th17 cytokine involved in protective immunity against many pathogens^40^, this did not appear to translate to increased efficacy of the intranasal NP vaccine. Th17 responses have been associated with autoimmune and inflammatory side effects^41^, but also with durability of vaccine-induced immune responses^42^. Potential risks and benefits of Th17 induction with NP-CpG vaccine still to be determined.

The intramuscularly administered alum-adjuvanted Spike-RBD vaccine, despite inducing a systemic humoral and Th2 cellular immune responses, failed to provide protection against SARS-CoV-2 infection. We attribute this to the use of monomeric RBD protein, which when compared to intramuscular immunization with full-length spike trimer induces significantly lower titers of neutralizing antibodies according to our earlier studies^22^. In general, the use of RBD protein in any COVID-19 vaccine, without the NP platform, requires large antigen doses and at least 3 immunizations to achieve protective efficacy^43^. Alum is a highly Th2-biased adjuvant, and in our earlier study^44^ with a veterinary COVID-19 vaccine called NARUVAX-C19 (pets) studied in juvenile cats, the alum adjuvant did not enhance neutralizing antibody titers compared to immunization with antigen alone.

Another important part of the present research was evaluation of the ability of the vaccines to protect against virus transmission from vaccinated challenged animals to naïve sentinels. Although transmission is influenced by many factors, the viral load in the upper respiratory tract is considered the best proxy for transmission risk^45^. Despite significantly lower titers of virus in nasal turbinates and oropharyngeal swabs of intranasal NP-immunized hamsters than controls, the NP-vaccines once infected still transmitted virus to sentinel animals placed in direct contact. This is consistent with other studies showing hamsters or humanized mice immunized with viral vector^46,47^ or genetic^48^ COVID-19 vaccines were still able to transmit infection to naïve animals. Human COVID-19 vaccines have also been observed to have an extremely modest or no effect on reducing the risk of virus transmission^49^, with similar peak virus loads observed in vaccinated and unvaccinated infected individuals^50^. Previously, our subunit spike-trimer based squalene emulsion-adjuvanted NARUVAX-C19 vaccine given IM protected against SARS-CoV-2 virus transmission in a hamster model, showing that blocking of transmission is achievable^22^. Many RBD-based vaccines require at least 3 doses to achieve maximum efficacy^51,52^, so it is possible we might have seen a greater effect on transmission if we had tested a 3-dose vaccine regimen. Transmission studies can be performed in different ways using either direct contact models or respiratory droplet/airborne transmission models where the animals are housed separately such that only the air is exchanged between animals. Both models provide informative data on different aspects of virus transmission, with transmission in the direct contact model presumably being much harder to prevent that transmission in a purely aerosol model. Hence we may still have seen vaccine effects on transmission if we had used a respiratory droplet/airborne transmission study apparatus, which was not available to us when we performed these studies.

We encountered certain limitations in our study due to constraints which prevented us from including larger group sizes or conducting repeated challenge and transmission studies. However, the immunogenicity data we obtained for the vaccines demonstrated consistency between mice and hamsters. While our vaccine was administered intranasally, based on the volumes administered studies have shown that some of the administered vaccine will also have also gone to the lungs^24^, so our results would represent the results of a combined intranasal and pulmonary immunization. This may have bearing on the fact that we saw less lung pathology in the intranasal NP vaccine groups than the IM RBD-alum group. This may affect the extrapolation of our results to larger animals and humans where the administered dose may be restricted solely to the nasal mucosa. Hence intranasal vaccines that are immunogenic and efficacious in rodents may not translate to comparable clinical immunogenicity/efficacy in humans^53^.

While the challenges in the current study were performed with a homologous virus in future studies, we intend to investigate the efficacy of our NP-vaccine in protecting against heterologous omicron virus variants. This would be important because the omicron strain is highly prevalent worldwide, and emerging evidence suggests that current IM COVID-19 vaccines offer suboptimal protection against omicron infections. We cannot exclude that the partial protection in the nasal turbinates and lungs observed with the intranasal NP vaccine might have been induced by innate immune responses or epigenetic reprogramming^54^, although we consider it more likely to have reflected a mucosal T cell response, even although this was not measured directly in the mucosa but just by performing cytokine recall responses on splenocytes. To exclude a role for innate immunity/epigenetic reprogramming, a control group dosed intranasally with a non-COVID protein conjugated to NP would need to be included as a control in future studies. Future studies will explore how the NP-vaccine can be modified to increase its systemic immunogenicity and protective efficacy, as reported in pigs vaccinated with chitosan-NP swine influenza vaccines^19,20^. We accept that the efficiency of incorporation of protein into our NP was low and the NP vaccine may have performed much better if this efficiency could be improved. Other strategies of NP immunization may also give better results such as a combination regimen where, for example our other subunit NARUVAX-C19 vaccine is administered IM followed by intranasal NP as a mucosal booster. It would also be interesting to test whether the efficacy of the NP vaccine could be improved by loading the NP with full spike trimer rather than just the RBD portion of the protein.

## Conclusion

An intranasally administered SARS-CoV-2 RBD-based nanoparticle vaccine induced sIgA and Th1-cell mediated immune responses in mice and provided protection against lung pathology and viral load in response to a homologous infection challenge in Syrian hamsters but failed to prevent virus replication in the nasal turbinates or contact-mediated virus transmission to co-housed naïve sentinel animals.

## Data Availability

Data are available from the corresponding author upon request.
