# Author Correction: An intranasal vaccine comprising SARS‑CoV‑2 spike receptor‑binding domain protein entrapped in mannose‑conjugated chitosan nanoparticle provides protection in hamsters

**DOI:** 10.1038/s41598-023-39818-8

**Published:** 2023-08-01

**Authors:** Kairat Tabynov, Maxim Solomadin, Nurkeldi Turebekov, Meruert Babayeva, Gleb Fomin, Ganesh Yadagiri, Sankar Renu, Toktassyn Yerubayev, Nikolai Petrovsky, Gourapura J. Renukaradhya, Kaissar Tabynov

**Affiliations:** 1 International Center for Vaccinology, Kazakh National Agrarian Research University (KazNARU), Almaty, Kazakhstan; 2 Preclinical Research Laboratory with Vivarium, M. Aikimbayev National Research Center for Especially Dangerous Infections, Almaty, Kazakhstan; 3 T&TvaX LLC, Almaty, Kazakhstan; 4 School of Pharmacy, Karaganda Medical University, Karaganda, Kazakhstan; 5 Central Reference Laboratory, M. Aikimbayev National Scientific Center for Especially Dangerous Infections, Almaty, Kazakhstan; 6 Center for Food Animal Health, College of Food Agricultural and Environmental Sciences, The Ohio State University (OSU), Wooster, OH 44691 USA; 7 Vaxine Pty Ltd, 11 Walkley Avenue, Warradale, SA 5046 Australia; 8 Republican Allergy Center, Research Institute of Cardiology and Internal Medicine, Almaty, Kazakhstan

Correction to: *Scientific Reports* https://doi.org/10.1038/s41598-023-39402-0, Published online 26 July 2023

The original version of this Article contained an error in the name of author Sankar Renu, which was incorrectly given as Renu Sankar.

The original Article has been corrected.

